# Genetic characterisation of multidrug-resistant *Salmonella enterica* serotypes isolated from poultry in Cairo, Egypt

**DOI:** 10.4102/ajlm.v4i1.158

**Published:** 2015-05-14

**Authors:** Mohammed Abdel-Maksoud, Rania Abdel-Khalek, Atef El-Gendy, Rawia F. Gamal, Hemmat M. Abdelhady, Brent L. House

**Affiliations:** 1 Global Disease Detection and Response Program, US Naval Medical Research Unit, Egypt; 2 Bacterial and Parasitic Disease Research Program, US Naval Medical Research Unit, Egypt; 3 Faculty of Agriculture, Ain Shams University, Department of Microbiology, Egypt

## Abstract

**Background:**

Food-borne diseases pose serious health problems, affecting public health and economic development worldwide.

**Methods:**

*Salmonella* was isolated from samples of chicken parts, skin samples of whole chicken carcasses, raw egg yolks, eggshells and chicken faeces. Resulting isolates were characterised by serogrouping, serotyping, antimicrobial susceptibility testing and detection of extended-spectrum β-lactamase (ESBL) production. Antibiotic resistance genes and integrons were identified by polymerase chain reaction (PCR).

**Results:**

The detection rates of *Salmonella* were 60%, 64% and 62% in chicken parts, skin, and faeces, respectively, whereas the egg yolks and eggshells were uniformly negative. *Salmonella* Kentucky and *S.* Enteritidis serotypes comprised 43.6% and 2.6% of the isolates, respectively, whilst *S.* Typhimurium was absent. Variable resistance rates were observed against 16 antibiotics; 97% were resistant to sulfamethoxazole, 96% to nalidixic acid and tetracycline and 76% to ampicillin. Multidrug resistance was detected in 82% (64/78) of the isolates and ESBL production was detected in 8% (6/78). The β-lactamase *bla*TEM-1 gene was detected in 57.6% and *bla*SHV-1 in 6.8% of the isolates, whilst the *bla*OXA gene was absent. The *sul1* gene was detected in 97.3% and the *sul2* gene in 5.3% of the isolates. Sixty-four of the 78 isolates (82%) were positive for the integrase gene (*int I*) from class 1 integrons, whilst *int II* was absent.

**Conclusion:**

This study reveals the presence of an alarming number of multidrug-resistant *Salmonella* isolates in the local poultry markets in Cairo. The high levels of drug resistance suggest an emerging problem that could impact negatively on efforts to prevent and treat poultry and poultry-transmitted human diseases in Egypt.

## Introduction

Food-borne diseases caused by non-typhoid *Salmonella* present an important public health problem that impacts significantly on the economy in many parts of the world. The main source of infection is food of animal origin, such as poultry, eggs, milk, beef and pork. In addition, fruits and vegetables have been implicated as vehicles for *Salmonella* transmission.^1^

Antibiotics are used extensively to prevent or treat microbial infections in veterinary medicine. Microbial infections may be detected at various levels in animal products and disseminated into the environment when manure is applied to fields.^2^ In the last two decades, antimicrobial resistance has emerged quickly amongst *Salmonella* isolates, creating a serious health hazard worldwide.^1^

Although fluoroquinolones have recently been used as the drug of choice to treat gastrointestinal infections in humans, resistant strains have since emerged and have been associated with increased illness and death.^3^ Whereas fluoroquinolones are contraindicated because of toxicity, cephalosporins have also been used to treat salmonellosis, particularly in children. However, resistance to this class of drugs appeared in 1992, mainly because of the emergence of extended-spectrum β-lactamases (ESBLs).^4^

ESBLs comprise rapidly evolving groups of β-lactamases, capable of hydrolysing (and thus inactivating) third-generation cephalosporins and aztreonam, which are inhibited by the β-lactamase inhibitor, clavulanic acid.^5^ The ESBLs are encoded frequently by genes located on R-plasmids, which often carry additional genes encoding resistance to other drug classes (e.g. fluoroquinolones and aminoglycosides).^6^ ESBL-producing strains of bacteria are emerging worldwide, particularly amongst the *Enterobacteriaceae*^7^ where the exchange of multidrug-resistant (MDR) plasmids between members of the family is common. This MDR can lead to severe limitations in treatment options for infection with these microorganisms, which are responsible for nearly half of all human infections.^8^

The presence of integron gene sequences has been identified as a primary method by which bacteria can acquire existing antimicrobial resistance genes. Each integron sequence is unique in that it acts as a site-specific recombination system capable of capturing or excising novel genetic elements called ‘gene cassettes’. These gene cassettes are promotorless genes with a recombination site known as a 59-base element or *att*C located at the 3′ end of the gene. The gene cassettes code for a wide range of antimicrobial resistant determinants.^9^ Only class 1 and 2 integrons have been detected in *Salmonella*, with class 1 being the most predominant.^10^

In Egypt, antibiotic resistance has been reported amongst human *Salmonella* isolates, including *Salmonella enterica* serovar Typhi (*S.* Typhi) and other diarrhoeagenic strains.^11,12^ However, with no national *Salmonella* surveillance centre to provide reliable statistical data, little is known about food-borne salmonellosis in Egypt. The present study was undertaken to determine the contamination rates of different *Salmonella* serotypes in chicken eggs, raw chicken meat and related environmental samples in poultry markets in Cairo, Egypt; and to characterise the identified isolates by serotype, antimicrobial susceptibility testing (AST) profiles and ESBL production. We also examined selected isolates to identify the presence of common antibiotic-resistance genes and integrons.

## Research methods and design

### Isolation and identification of *Salmonella* from poultry meat, egg and faecal samples

A total of 165 samples were collected between December 2011 and May 2012 from 18 poultry markets (mostly street markets and retail shops that sell meat and live birds) distributed throughout 5 geographical locations in Cairo Governorate. The food samples were collected from 62 chicken meat parts (20 boneless breasts, 19 cloacae, 10 livers, 8 gizzards and 5 wings), 22 skin pieces from slaughtered birds, 30 raw egg yolks and shells from another 30 eggs. The faecal samples were collected from 21 separate chicken faeces specimens. The chicken parts and carcass samples were taken from different birds; all samples were cultured within 2 hours. The faecal samples were obtained from the same 18 poultry markets.

*Salmonella* strains were isolated and identified according to standard methods,^13^ and colonies that exhibited typical biochemical reactions were further confirmed as *Salmonella* using the API 20E identification kit (Biomerieux, Craponne, France).

### Serogrouping and serotyping of *Salmonella* isolates

Biochemically identified *Salmonella* isolates were serogrouped initially by slide agglutination using commercially-available *Salmonella* O antiserum (Difco Laboratories, Detroit, MI, United States). Because of funding limitations, only serogroup B, C2 and D isolates were then serotyped for *S*. Typhimurium, *S.* Enteritidis and *S.* Kentucky according to the Kauffman White scheme,^14^ using *Salmonella* H antisera (Sifin, Germany; Statens, Denmark).

### Antibiotic susceptibility testing and detection of extended-spectrum β-lactamase production

Antimicrobial susceptibilities to tetracyclines (tetracycline), sulphonamides (sulfamethoxazole trimethoprim/sulphamethoxazole), quinolones (nalidixic acid), penicillins (ampicillin), penicillin/β -lactamase inhibitor combinations (ticarcillin/clavulanate, ampicillin/sulbactam), phenicols (chloramphenicol), fluoroqinolones (ciprofloxacin), aminoglycosides (streptomycin, gentamicin, amikacin), monobactams (aztreonam), cephalosporins (cefotaxime, ceftriaxone, ceftazidime, cefepime) and carbapenems (imipenem) were determined using Kirby-Bauer disc diffusion according to Clinical and Laboratory Standards Institute (CLSI)^15^ guidelines. In addition, minimum inhibitory concentration (MIC) was determined using *E*-test methods (AB Biodisk, Solana, Sweden), also according to CLSI guidelines. MDR *Salmonella* was defined as any isolate that showed resistance to at least three different classes of antibiotics.^11^

Screening for ESBL production in *Salmonella* isolates was done using the standard procedure of measuring the zones of inhibition surrounding cefotaxime and ceftazidime discs versus cefotaxime-clavulanic acid and ceftazidime-clavulanic acid discs, respectively. Any isolate with a ≥ 5 mm increase in zone diameter for either antibiotic tested in combination with clavulanic acid, versus without clavulanic acid, was considered to be an ESBL producer.^15^ The reference strains *Escherichia coli* ATCC 25922 and *Staphylococcus aureus* ATCC 25923 were used to verify the quality and accuracy of testing procedures.

### Detection of antimicrobial resistance genes

*Salmonella*-isolate DNA was purified using the DNA-boiling method suggested by Sambrook, Fritsch and Maniatis.^16^. The following genes implicated in antimicrobial resistance were detected by PCR amplification: for β-lactam resistance – *bla*TEM-1, *bla*SHV-1 and *bla*OXA-1; for sulphonamide resistance – *sul1* and *sul2*. The primer sets and assay conditions used for amplification were as described previously.^17,18^

### Detection and characterisation of integrons

The presence of class 1 and 2 integrase-coding genes (*int I* and *int II*) were detected by PCR with specific primers.^17,19^ Primers 5’-conserved segment (CS) and 3’-CS described by Levesque,^20^ targeting the inserted gene cassette regions of class 1 integrons, were used to determine these regions.

### Statistical analysis

For statistical analyses to detect significant differences between antibiotic resistance rates, *p* values were determined by using Student's *t*-test in Microsoft® Office Excel 2010 (Microsoft Corp., Redmond, WA, United States).

## Results

*Salmonella* isolates were recovered from 64 of the 165 samples collected, including 60% of chicken meat samples, 64% of chicken carcasses (skin) samples and 62% of chicken faeces samples. No *Salmonella* was isolated from raw egg yolk or eggshell samples.

Serogroups identified amongst the S*almonella* isolates were B, C1, C2, and D. Fifty-one samples yielded isolates from one serogroup and 12 samples yielded isolates from 2 serogroups, whilst one sample from skin yielded 3 serogroups. Of the 78 *Salmonella* isolates obtained, group C2 was the predominant group found in chicken meat (Table 1). Serotypes identified included *S.* Kentucky (43.6%) and *S.* Enteritidis (2.6%) (Table 2). *S.* Typhimurium was not identified.

**TABLE 1:** Distribution of *Salmonella* serogroups isolated from 144 poultry samples and 21 faecal samples collected between December 2011 and May 2012.

*Salmonella* serogroups	No. of isolates in each serogroup (% of sample type)		
	Chicken meat and skin samples†	Faecal samples‡	Total
Group B	12 (18.5)	3 (23)	15 (19.2)
Group C1	21 (32.3)	2 (15)	23 (29.5)
Group C2	30 (46.2)	8 (62)	38 (48.7)
Group D	2 (3)	–	2 (2.6)
Total	65	13	78

†, A total of 144 samples were collected from 62 chicken meat parts (20 boneless breasts, 19 cloacae, 10 livers, 8 gizzards and 5 wings), 22 skin pieces from slaughtered birds, 30 raw egg yolks and shells from another 30 eggs.

‡, A total of 21 samples were collected from chicken faeces.

**TABLE 2:** Distribution of serotypes for the 78 *Salmonella* isolates recovered from poultry and faecal samples collected between December 2011 and May 2012.

*Salmonella* serotypes	No. of isolates in each serotype (% of sample type)		
	Chicken meat and skin samples†	Faecal samples‡	Total
S. Kentucky	27 (41.5)	7 (54)	34 (43.6)
S. Enteritidis	2 (3.1)	–	2 (2.6)
S. Typhimurium	–	–	-
Other serotypes§	36 (55.4)	6(46)	42 (53.8)

†, A total of 144 samples were collected from 62 chicken meat parts (20 boneless breasts, 19 cloacae, 10 livers, 8 gizzards and 5 wings), 22 skin pieces from slaughtered birds, 30 raw egg yolks and shells from another 30 eggs.

‡, A total of 21 samples were collected from chicken faeces.

§, Further serotyping was not performed for these isolates because of funding limitations.

Overall, there was a high level of antibiotic resistance found amongst the *Salmonella* isolates (Table 3). Resistance was detected to 16 out of 18 antibiotics tested, whilst all *Salmonella* isolates were susceptible to imipenem and cefepime. Isolates demonstrated high levels of resistance to sulfamethoxazole (97%), nalidixic acid and tetracycline (96%), ampicillin (76%), ticarcillin/clavulanate (67%), chloramphenicol (56%) and ciprofloxacin (46%). Multidrug resistance was observed amongst 82% (64/78) of the isolates, with 59 isolates (76%) resistant to more than 5 antibiotics. ESBL production was detected in 8% (6/78), with all 6 being highly resistant to multiple antibiotics when compared to non-ESBL producing strains. When compared with other *Salmonella* serotypes, the *S*. Kentucky isolates showed higher resistance rates to the majority of antibiotics tested, reaching statistical significance against ciprofloxacin and ticarcillin/clavulanate (*p* < 0.01). Sixteen (46%) *S.* Kentucky strains were resistant to at least 8 antibiotics.

**TABLE 3:** Percent antibiotic resistance and MIC range of *S.* Kentucky, other *Salmonella* serotypes and ESBL-producing *Salmonella* isolates from poultry meat and faecal samples.

Class and antibiotics (μg/disc)	*E*-test	Disk diffusion: No. of samples demonstrating resistance (percentage of isolates resistant)			
	*MIC Range* μg/mL	*Salmonella* Kentucky (*n* = 32)†	Other *Salmonella* serotypes (*n* = 40)	*Salmonella* ESBL-producers (*n* = 6)	Total (*n* = 78)
**Sulphonamides**					
Sulfamethoxazole (300 μg)	NA	32 (100)	38 (95)	6 (100)	76 (97)
Trimethoprim/Sulphamethoxazole (1.25 μg/23.75 μg)	0.094 – >32	23 (72)	14 (35)	3 (50)	40 (51)
**Tetracyclines**					
Tetracycline (30 μg)	2 – >256	31 (97)	38(95)	6(100)	75(96)
**Quinolones**					
Naldixic acid (30 μg)	4 – >256	30 (94)	39 (98)	6 (100)	75 (96)
**Penicillins**					
Ampicillin (10 μg)	24 – >256	31 (97)	22 (55)	6 (100)	59 (76)
**β-lactamases**					
Ticarcillin/clavulanate (75/10 μg)	NA	30 (94)‡	17 (43)‡	5 (83)	52 (67)
Ampicillin/sulbactam (10/10 μg)	3-256	11 (34)	12 (30)	2 (33)	25 (32)
**Phenicols**					
Chloramphenicol (30 μg)	NA	26 (81)	16 (40)	2 (33)	44 (56)
**Fluoroquinolones**					
Ciprofloxacin (5 μg)	0.19–12	31 (97)‡	1 (3)‡	4 (66)	36 (46)
**Aminoglycosides**					
Streptomycin (10 μg)	0.38–512	1 (3)	21 (53)	6 (100)	28 (36)
Gentamicin (10 μg)	0.38–24	9 (28)	13 (33)	3 (50)	24 (31)
Amikacin (30 μg)	1–2	1 (3)	0	0	1 (1)
**Monobactam**					
Aztreonam (30 μg)	NA	1 (3)	0	6 (100)	7 (9)
**Cephems**					
Cefotaxime (30 μg)	24 – >256	1 (3)	1 (3)	6 (100)	8 (10)
Ceftriaxone (30 μg)	0.125 – >32	1 (3)	0	4 (66)	5 (6)
Ceftazidime (30 μg)	0.38 – >256	1 (3)	0	4 (66)	5 (6)
Cefepime (30 μg)	0.19–3	0	0	0	0
**Carbapenems**					
Imipenem (10 μg)	0.038-0.125	0	0	0	0

†, 2 of the 34 *Salmonella* Kentucky isolates were ESBL-producers and are included in the *Salmonella* ESBL producers column.

‡, Significant difference in resistance level found between *S.* Kentucky and other *Salmonella* serotypes were calculated at *p* < 0.01.

MIC, minimum inhibitory concentration; ESBL, extended-spectrum β-lactamases.

Amongst the 75 strains resistant to nalidixic acid, 36 were resistant to the related ciprofloxacin (MIC range of 4–12 μg/mL). Imipenem showed the lowest MIC values, followed by cefepime and amikacin, all of which were found to be effective against *Salmonella* strains isolated from poultry in Cairo. On the other hand, the highest MIC values were obtained against streptomycin, followed by nalidixic acid and ampicillin (Table 3).

Table 4 lists the resistance genes detected in the *Salmonella* isolates. The most frequent β-lactam gene identified amongst ampicillin resistant isolates was *bla*TEM-1, detected in 57.6% of the isolates, followed by *bla*SHV-1 which was identified in 6.8%. The *bla*OXA-1 gene was not detected in any isolate in this study. Regarding sulphonamide resistance genes, the presence of the *sul1* gene was detected in 97.3% of the isolates and the *sul2* gene in 5.3%. Four isolates possessed both the *sul1* and *sul2* genes (Table 4).

**TABLE 4:** Antimicrobial resistance genes and the resistance phenotype of *S. enterica* strains isolated from poultry meat and faecal samples (*n* = 78).

Class and antimicrobial	No. of resistant isolates (%)	Resistance gene	Isolates containing the selected resistance gene			
			No. (%)	Serovar distribution (no. of isolates)	Origin and no. of isolates	
					Poultry meat	Faecal samples
β-Lactams Ampicillin	59 (75.6)	blaTEM-1	34 (57.6)	S. Kentucky (22), other serotypes (10)	30	4
		blaOXA-1	0	–	–	–
		blaSHV-1	4 (6.8)	Other serotypes (4)	4	–
		Others	21 (35.6)	S. Kentucky (9), other serotypes (12)	16	5
Sulphonamides Sulfamethoxazole	76 (97.4)	sul1	74 (97.3)	S. Kentucky (35), other serotypes (39)	61	13
		sul2	4 (5.3)	S. Kentucky (1), other serotypes (3)	4	–
		Others	2 (2.6)	Other serotypes (2)	2	–

Sixty-four of the 78 isolates (82%) were positive for the *int I* gene, whilst *int II* was absent. The 5′- and 3′-CS regions were identified in 46.8% of the *int I* positive isolates. Eight types of class 1 integrons were detected for the *Salmonella* spp. isolates, including the 1950 bp, 1550 bp, 1200 bp, 1100 bp, 1000 bp, 700 bp, a combination of 950 bp and 1200 bp and a combination of 1100 bp and 1550 bp integrons. Five *S.* Kentucky isolates resistant to ciprofloxacin carried the 1950 bp class 1 integron (Table 5).

**TABLE 5:** Characteristics of class 1 integron-carrying multidrug-resistant *S. enterica* strains isolated from poultry meat and faecal samples (*n* = 64).

Amplicon size in class 1 integron-PCR (bp)	No. (%)	Resistance genes	Antibiotic resistance profile†	ESBL	Serogroup (no. of isolates)	Serotype‡	Origin and no. of isolates	
							Poultry meat	Faecal samples
700	2 (3.1)	*sul1, bla*TEM-1	SUL, NA, TE, TIM, C, SXT, SAM	–	B (1), C2 (1)	N/A	2	–
700	2 (3.1)	*sul1, bla*TEM-1, blaSHV-1	SUL, NA, TE, AM, S, TIM, SXT, CTX, ATM, CAZ	+	B (2)	N/A	2	–
950–1200	1 (1.6)	*sul1*	SUL, NA, TE, S, TIM, SXT	–	C1 (1)	N/A	1	–
1000	10 (15.6)	*sul1, sul2, bla*TEM-1	SUL, NA, TE AM	–	C1 (9), C2 (1)	N/A	8	2
1100	2 (3.1)	*sul1*	SUL, NA, TE S, TIM, SXT	–	B (2)	N/A	1	1
1100–1550	4 (6.3)	*sul1, bla*TEM-1	SUL, NA, TE AM, S, GM, SAM	–	B (4)	N/A	4	–
1200	2 (3.1)	*sul1, sul2, bla*TEM-1	SUL, NA, TE AM	–	C1 (2)	N/A	2	–
1550	2 (3.1)	*sul1, bla*TEM-1	SUL, NA, TE AM S, GM, CIP	–	C2 (2)	S. Kentucky	2	–
1950	5 (7.8)	*sul1, bla*TEM-1	SUL, NA, TE, CIP	–	C2 (5)	S. Kentucky	4	1
No amplicon	30 (46.9)	*sul1, sul2, bla*TEM-1, blaSHV-1	SUL, NA, TE	–	B (5), C1 (6), C2 (19)	S. Kentucky (18), N/A (12)	26	4
No amplicon	4 (6.3)	*sul1, sul2, bla*TEM-1, blaSHV-1	SUL, NA, TE AM, S, CTX, ATM	+	B (2), C2 (2)	S. Kentucky (1), N/A (2)	3	1

‡, N/A: not applicable.

†, Antibiotics listed in the antibiotic profile are those that demonstrated 100% resistance with all tested isolates.

AM, Ampicillin; ATM, Azetronam; C, Chloramphenicol; CAZ, Ceftazidime; CIP, Ciprofloxacin; CTX, Cefotaxime; GM, Gentamicin; NA, Nalidixic acid; S, Streptomycin; SAM, Ampicillin/sulbactam; SUL, Sulfonamide compounds; SXT, Sulfamethoxazole/Trimethoprim; TE, Tetracycline; TIM, Ticarcillin clavulanate.

## Discussion

The objectives of this study were to determine the frequency of *Salmonella* contamination of chicken eggs, meat and faeces in poultry markets in Cairo, Egypt and to identify prevalent serotypes and antibiotic susceptibility profiles, including ESBL production. The results from this study revealed levels of *Salmonella*-contamination in fresh chicken meat of 60% – 64%, which are higher than those previously reported in Assiut, Egypt from frozen chicken legs and fillet samples (36% – 52%);^21^ in Senegal from chicken carcasses (32%);^22^ and in Ethiopia from raw chicken meat and giblets (18%).^23^ In contrast to a study conducted by Del Cerro et al.^24^ which reported that faeces from chickens were positive for *Salmonella* by culture in 39% of the samples tested, our study demonstrated a 62% positivity rate for *Salmonella* isolation from faecal specimens. The high contamination rates seen in this study may, at least in part, be explained by the lack of hygienic slaughtering processes that occur commonly at small shops, away from the modern abattoirs available for mass slaughtering of poultry. At these shops, slaughtering is manual, rudimentary and may take place either indoors or outdoors. Usually, one person provides all the labour, including live bird care, cleaning, slaughtering, de-feathering and evisceration, increasing the likelihood of cross-contamination amongst birds.

In this study, serogroups B, C1 and C2 accounted for 97% of the isolates from chicken meat. A similar study performed in the Pacific Northwest, in the United States^25^ also found that serogroups B and C comprised the majority (95%) of all *Salmonella* isolated from poultry and the poultry environment. A study in Saudi Arabia^26^ reported that 64% of the isolates from poultry and the poultry environment were from groups B and C. In the current study, there were only two isolates assigned to serogroup D, subsequently serotyped as *S*. Enteritidis.

In humans, *S.* Enteritidis and *S.* Typhimurium have been reported to be the two most prevalent *Salmonella* serotypes in many regions of the world.^26^ In addition, a study in Turkey demonstrated that *S.* Enteritidis was the most prevalent *Salmonella* serotype isolated from chicken meat.^27^ However, a study in Senegal identified only 6 *S*. Enteritidis serotypes out of 90 *Salmonella* strains isolated.^28^ Another study in Sudan identified 2 chicken and 2 human origin *S*. Kentucky strains resistant to both ciprofloxacin and norfloxacin out of 64 *Salmonella* isolates studied.^29^ Interestingly, studies in France,^30^ Switzerland^31^ and the Slovak Republic^32^ have reported that infection with *S*. Kentucky strains resistant to ciprofloxacin was associated with travel to Egypt, Morocco and other countries in the North African region. Corroborating these studies, our results indicate that the most prevalent serotype in Cairo, Egypt is *S.* Kentucky, with high rates of resistance to ciprofloxacin. *S.* Enteritidis was isolated at a very low rate, and *S.* Typhimurium was not detected at all. This is comparable to the results from Senegal,^27,29^ where *S*. Kentucky was also found to be the most prevalent serotype (30% of the total isolates). The observed low isolation rates for *S.* Enteritidis and *S.* Typhimurium in this study may result from replacement by other serotypes (54% were not serotyped in this study).

The remarkably high rates of antibiotic resistance exhibited by *Salmonella* strains from this study, particularly against sulfamethoxazole (97.4%), nalidixic acid (96.2%), tetracycline (96.2%), ampicillin (75.6%) and streptomycin (35.9%), are probably because of the early introduction and consequent widespread use of these antibiotics in veterinary and human medicine in Egypt. The high resistance rates to nalidixic acid and ciprofloxacin are of particular note, since quinolones have been considered one of the last options for the treatment of MDR *Salmonella*.

All *S*. Kentucky strains tested in this study were MDR and demonstrated significantly higher rates of resistance than other *Salmonella* serotypes to ciprofloxacin (97% vs. 2.5%; *p* < 0.01) and ticarcillin/clavulanate (94% vs. 42.5%; *p* < 0.01). The correlation between antibiogram and serotype suggests poor infection control practices in the poultry production industry, which may facilitate the spread of these MDR *S.* Kentucky strains.

The most common mode of bacterial-acquired resistance to β-lactam antibiotics is the β -lactamase enzyme.^34^ In this study, 30 of 48 poultry meat isolates and 4 of 11 faeces isolates that were ampicillin resistant possessed the *bla*TEM gene. This result is in agreement with other findings.^1,17^ Recently, ESBL acquisition rates by *Salmonella*, in particular, have arisen worldwide. In an earlier study conducted on *Salmonella* isolates from poultry in Egypt, 5% of the isolates belonging to serovar Poona produced ESBLs.^12^ In this study 8% (*n* = 6) of *Salmonella* isolates demonstrated ESBL production, two of them belonging to serovar Kentucky. The detection of an ESBL phenotype in poultry meat and faecal samples in this study may indicate a lack of infectious disease barriers amongst clinics, humans and animals. In addition, approximately half of all antibiotics produced worldwide (many of which are used routinely in humans) are used in animals to prevent infection and consequently improve production.^35^ Unfortunately, this leads to the development of resistant bacteria in animals that can infect humans directly or transfer antibiotic resistance genes to other human pathogens.^36^

Sulphonamides are amongst the most commonly-used antibiotics for food animal production worldwide.^37^ These compounds are bacteriostatic antimicrobial drugs that act by means of competitive inhibition of the enzymes involved in the synthesis of tetrahydrofolic acid. Sulphonamides compete with the structural analogue *p*-aminobenzoic acid binding to dihydropteroate synthetase (DHPS), a catalytic enzyme in the folic acid biosynthesis pathway, thus inhibiting the formation of dihydrofolic acid.^38^ Sulphonamide resistance in *Salmonella* isolates has been attributed to the presence of an extra *sul* gene, which expresses an insensitive form of DHPS. In this study, the presence of the *sul1* gene was detected in 74 of 76 sulphonamide-resistant isolates. The PCR results were consistent with the antimicrobial susceptibility phenotypes; the *sul1* and/or *sul2* genes were detected in 97.4% of the sulphonamide-resistant *Salmonella* isolates. Other studies have found this gene to be present at moderate to high rates in retail meats and other foods.^1,17,39^

Integrons are genetic elements that are able to recognise and capture mobile gene cassettes carrying the antibiotic resistance genes, which leads to MDR distribution and the subsequent limitation of treatment options for infectious diseases.^40^ In this study, PCR screening results of 78 *Salmonella* isolates detected class 1 integrons in 62 (79.5%) isolates. Very few studies have investigated class 1 integrons in *Salmonella* isolates from human, poultry and faeces isolates in Egypt. The presence of integrons was examined in 21 *Salmonella* isolates from diseased broiler chickens in Egypt where the researchers identified class 1 and class 2 integrons in 42.9% and 14.3% of the isolates, respectively.^41^ Lower detection rates were obtained in a study of food isolates in Germany (65%).^1^ A study by Antunes, Machad and Peixe^42^ showed, in a large survey of 1183 *Salmonella* isolates from various animal, human and food sources, that 75% carried class 1 integrons. Class 1 integrons have been detected in *S*. Kentucky.^43^ In our study, 5 isolates of *S*. Kentucky carried the 1950 bp class 1 integron.

Multidrug resistance was observed amongst 82% (64/78) of the isolates, with 59 isolates (76%) being resistant to more than 5 antibiotics. Our study indicates that 88.1% of the 59 MDR isolates harboured class 1 integrons, whilst none of the MDR isolates carried class 2 integrons. Several groups have reported that integron-containing isolates are more antibiotic resistant than those isolates obtained from comparable patients which were lacking an integron.^44^

## Limitations

Samples included in this study were collected from the Cairo Governorate during the six-month period from December 2011 to May 2012. Thus, the results of this study may not be generalisable to other regions or seasons. More studies are needed on samples collected from the Nile Delta and Upper Egypt governorates and during different seasons.

## Conclusion

In conclusion, this study demonstrated a relatively high prevalence of *Salmonella*-contaminated poultry products, with *S*. Kentucky the most prevalent serotype, in poultry markets in Cairo, Egypt. In addition, this study revealed significant MDR rates, particularly carried by *S*. Kentucky serovar strains, against the β-lactam and fluoroquinolone (e.g., ciprofloxacin) classes of antibiotics. Ultimately, these trends may limit treatment options and contribute to treatment failure and increased death rates.

More comprehensive studies are needed to better determine the prevalence and antibiotic resistance patterns of *Salmonella-*contaminated poultry meat and its products. More serotypes should be utilised in identification and be included in a national surveillance database to allow comparisons with findings within Egypt and from other countries in the region. This surveillance should include antimicrobial susceptibility profiles to track the emergence and exacerbation of existing drug resistance amongst *Salmonella* and other food-borne disease pathogens.

## Copyright assignment statement

The authors are employees of the US Government. This work was prepared as part of their official duties. Title 17 U.S.C. §105 provides that ‘Copyright protection under this title is not available for any work of the United States Government.’ Title 17 U.S.C. §101 defines a US Government work as a work prepared by a military service member or employee of the US Government as part of that person's official duties.
